# Regression Modeling of ZAP-X Treatment Time

**DOI:** 10.7759/cureus.86748

**Published:** 2025-06-25

**Authors:** Michael Chaga, Akil Anthony, Timothy Chen, Wenzheng Feng, Tingyu Wang, Darra Conti, Jing Feng, Ma Rhudelyn Rodrigo, Patrick Pema, Elizabeth Luick, Daniel Thompson, Joy Baldwin, Brielle Latif, Georgia Montone, Joseph Hanley, Shabbar Danish

**Affiliations:** 1 Radiation Oncology, Jersey Shore University Medical Center, Neptune, USA; 2 Genetics and Statistics, Rutgers University, New Brunswick, USA; 3 Neurosurgery, Hackensack Meridian Health, Hackensack, USA; 4 Neurosurgery, Jersey Shore University Medical Center, Neptune, USA

**Keywords:** regression modeling, srs, stereotactic radiosurgery, treatment time, zap-x

## Abstract

Accurate prediction of treatment time is critical in stereotactic radiosurgery (SRS) for optimizing patient scheduling and workflow efficiency. The ZAP-X system (ZAP Surgical Systems, Inc., San Carlos, CA), the newest cranial SRS platform, provides a built-in estimate of treatment time but often underestimates the actual treatment duration. This study aimed to develop a robust predictive model for ZAP-X treatment time, improving accuracy and reliability.

Prospective data of 200 patients treated with ZAP-X SRS at Jersey Shore University Medical Center including timing metrics, treatment planning variables, and clinical factors were analyzed. Random Forest regression identified the most critical factors influencing treatment time. Subsequently, Ridge Regression was applied to develop a predictive model, with 10-fold cross-validation used for model tuning and validation.

Random Forest analysis identified setup time and gantry time as the most influential variables affecting the treatment duration, followed by the number of beams, number of isocenters, dose, and target number. The Ridge Regression model demonstrated strong predictive performance (R² = 0.984, MAE = 1.94 minutes, RMSE = 2.49 minutes), significantly surpassing the ZAP-X system’s internal estimates. The model’s accuracy can be attributed to its ability to account for procedural variability, particularly in setup time.

The Ridge Regression model provides a highly accurate and interpretable method for predicting ZAP-X treatment time, outperforming the system’s internal calculations. This model has immediate clinical utility for improving patient scheduling and resource management in SRS. Future work should focus on validating the model across institutions and adapting it to evolving ZAP-X technology.

## Introduction

The ZAP-X (ZAP Surgical Systems, Inc., San Carlos, CA) is the newest dedicated cranial stereotactic radiosurgery (SRS) platform. The radiation source for the ZAP-X is a 3 MV S-band linear accelerator (Linac) mounted on axial and oblique gimbals that allows for isocentric, non-coplanar, dual-axis radiation delivery over a solid angle of greater than 2π steradians. The self-shielded ZAP-X system design reduces radiation exposure to low levels inside the treatment room, eliminating the need for a shielded vault and increasing installation flexibility [1-3]. 

Analysis of this newly pioneered system is critical to gauge its efficacy and refine its specificity for varying patients and conditions. Various parameters are employed to facilitate this, including SRS treatment time. In Radiation Oncology, SRS treatment time can be defined as the total time for patient setup, imaging, and radiation delivery for a treatment session. The setup and imaging time encompasses the duration required for positioning the patient, delivering the imaging, and evaluating and applying the necessary table corrections. The treatment time can vary based on the planning technique and treatment machine used. For example, complex treatment plans typically require longer delivery times, including extended gantry or couch movements [4]. Evaluating and predicting treatment time is critical for patient comfort and scheduling in the Radiation Oncology setting. Forecasting in healthcare can be achieved through qualitative or quantitative methods. Qualitative methods, like the Delphi method, depend on expert opinions, while quantitative techniques, such as time series forecasting, utilize historical data for future predictions [5,6]. In cancer care, time series methods like regression analysis, autoregressive integrated moving average, and machine learning are frequently employed to forecast cancer incidence [6-12]. In this study, regression modeling of ZAP-X treatment time is performed to identify the most important factors and accurately predict treatment time.

## Technical report

Data collection and processing

This study used prospective data from 200 patients treated with ZAP-X SRS at Jersey Shore University Medical Center (JSUMC). For patients with multiple treatment sessions, all data were averaged per patient, so the final dataset reflects patient-level treatment profiles. This reduced variability from session-level anomalies while maintaining individual treatment characteristics. Statistical analysis was conducted in R version 4.4.1 and Stata 18 (Release 18; StataCorp LLC, College Station, TX) on the variables presented in Table 1. Timing metrics for each patient were obtained from reports generated by the ZAP-X system upon completion of treatment. Treatment time is the duration from initial auto-alignment to the end of treatment. Calculated treatment time is the predicted treatment duration by the ZAP-X treatment planning system (TPS). Setup time is the auto-alignment imaging and review duration. Gantry time is the duration for the gantry to move all gantry angles throughout the entire delivery. Table time is the duration for the treatment table to move to all table locations throughout the entire delivery. kV imaging and processing time is the duration to acquire the kV images throughout the entire delivery, match to digitally reconstructed radiographs, and perform noise reduction, edge detection, and normalization. Linac time is the beam on delivery duration. Treatment planning variables were obtained from Eclipse TPS (Varian Medical Systems, Palo Alto, CA, version 15.6) and ZAP-X TPS (versions 1.8 - 1.10). Clinical variables were obtained from Aria Electronic Medical Records (Varian Medical Systems, Palo Alto, CA).

**Table 1:** Timing metrics, treatment planning, and clinical variables used for statistical analysis.

Category	Variable
Timing Metrics	Treatment time, calculated treatment time, setup time, gantry time, table time, kV imaging and processing time, Linac time
Treatment Planning	Target number, target volume, dose, prescription isodose line, fractions, number of beams, number of isocenters, total collimator size, total path number, target coverage, conformity index, gradient index, homogeneity index, gradient measure, monitor units
Clinical	Pathology (numerically encoded: Brain Metastases = 1, Meningioma = 2, Trigeminal Neuralgia = 3, Acoustic Neuroma = 4, Recurrent Glioblastoma = 5, Pituitary Adenoma = 6, Spinal Tumor = 7, Arteriovenous Malformation = 8, Hemangioma = 9), Eastern Cooperative Oncology Group score, age

Initial observation

**Figure 1:**
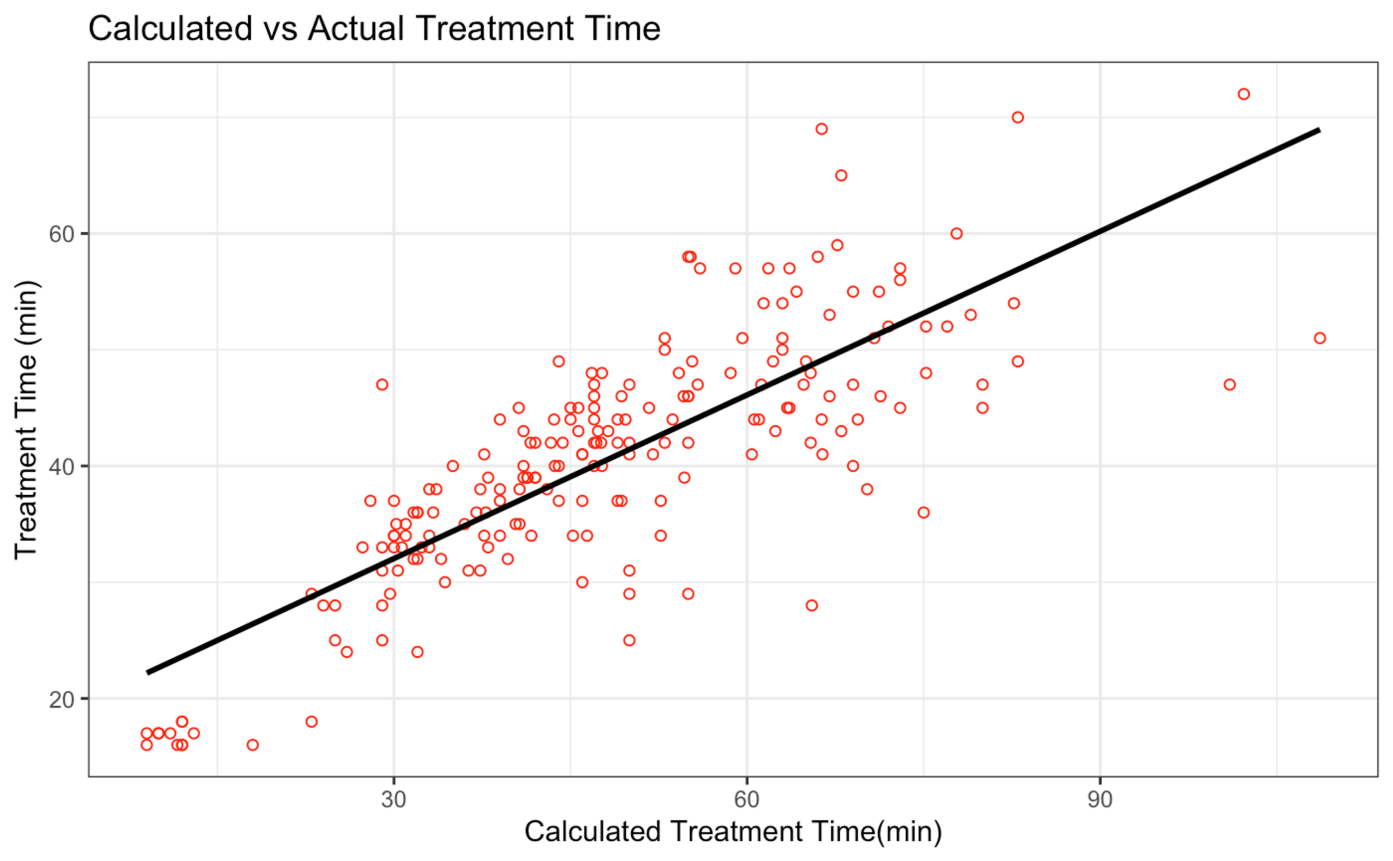
A scatterplot comparing the ZAP-X calculated treatment time to the actual treatment time recorded for each patient. The regression line (black) shows that the system consistently underestimates real-world treatment durations, highlighting the need for a more accurate predictive model. Image Credit: Akil Anthony

A simple linear model comparing ZAP-X calculated and actual treatment time yielded (Figure 1): (i) Adjusted coefficient of determination (R²) = 0.64: Suggesting that only 64% of the variation in real treatment time could be explained by the machine's own estimate, (ii) Root Mean-Squared Error (RMSE) = 11.12 minutes: On average, predictions were within 11.12 minutes of the real value, with larger errors penalized more heavily, and (iii) Mean Absolute Error (MAE) = 8.30 minutes: On average, predicted times were 8.30 minutes off from actual times. This gap demonstrates the need for a better predictive model.

Variable importance: Random Forest

**Figure 2:**
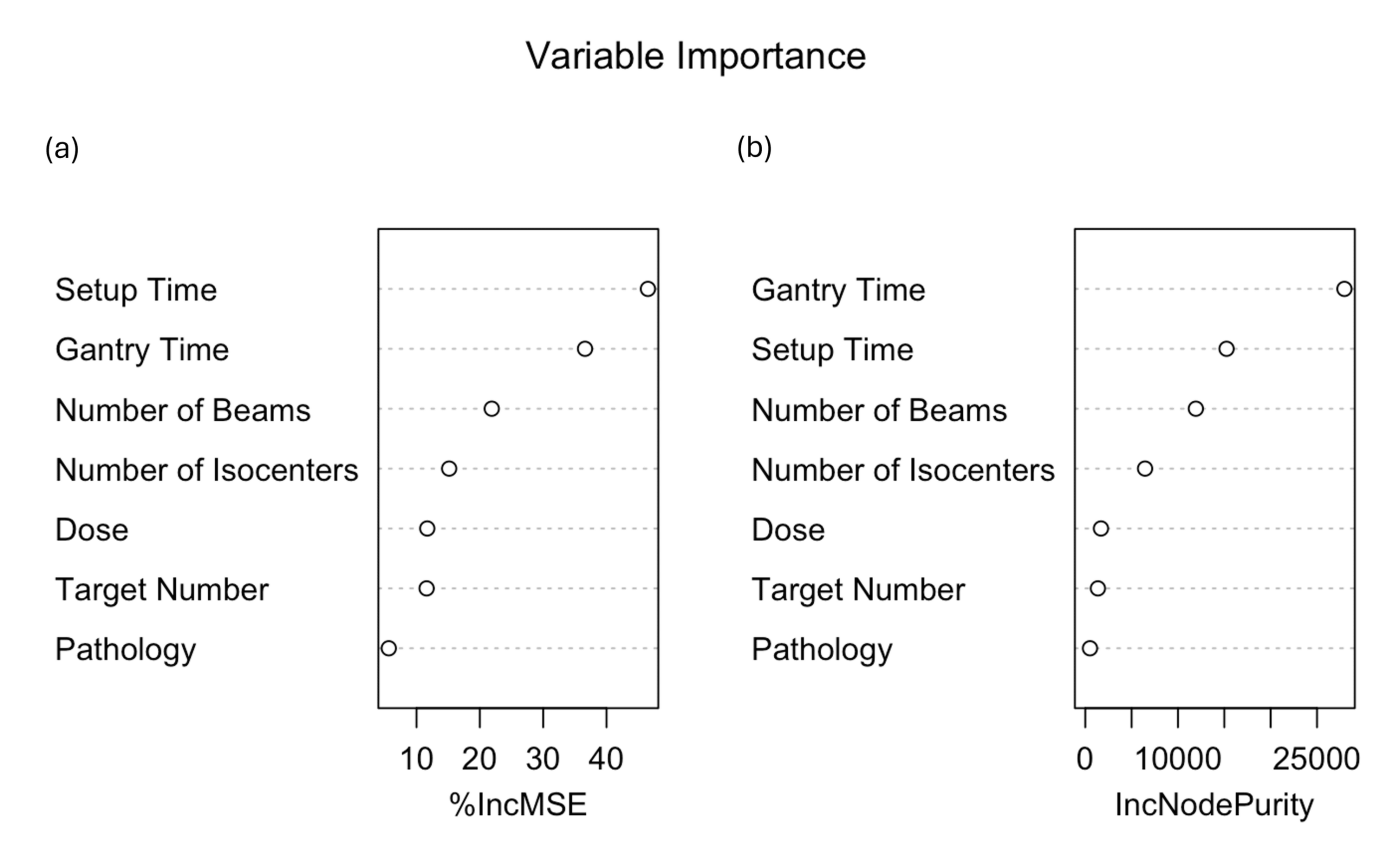
Variable importance plots from the Random Forest model: (a) Percentage increase in mean-squared error (%IncMSE), which measures how much prediction error increases when a variable is randomly permuted — higher values indicate more predictive power. (b) Increase in node purity (IncNodePurity), a measure of how much each variable contributes to reducing variance within decision tree splits. Image Credit: Akil Anthony

We used a Random Forest regression model to explore which factors most impact actual treatment time. Random Forests are machine learning models that generate many decision trees and average their predictions. Such models are useful because they handle nonlinear relationships and interactions between variables without the need for manual specification [13]. Key results from our Random Forest model are as follows: (i) R² = 0.920: The model explained 92% of the variance in actual treatment time, (ii) RMSE = 5.52 minutes: On average, predictions were within 5.52 minutes of the real value, with larger errors penalized more heavily, and (iii) MAE = 3.73 minutes: On average, predicted times were 3.73 minutes off from actual times. The most important predictors were setup time and gantry time, followed by the number of beams, number of isocenters, dose, and target number. However, setup time is the most influential, indicating that the ZAP-X's time estimate likely overlooks the variability introduced by the patient setup (Figure 2).

Predictive modeling: Ridge Regression

**Figure 3:**
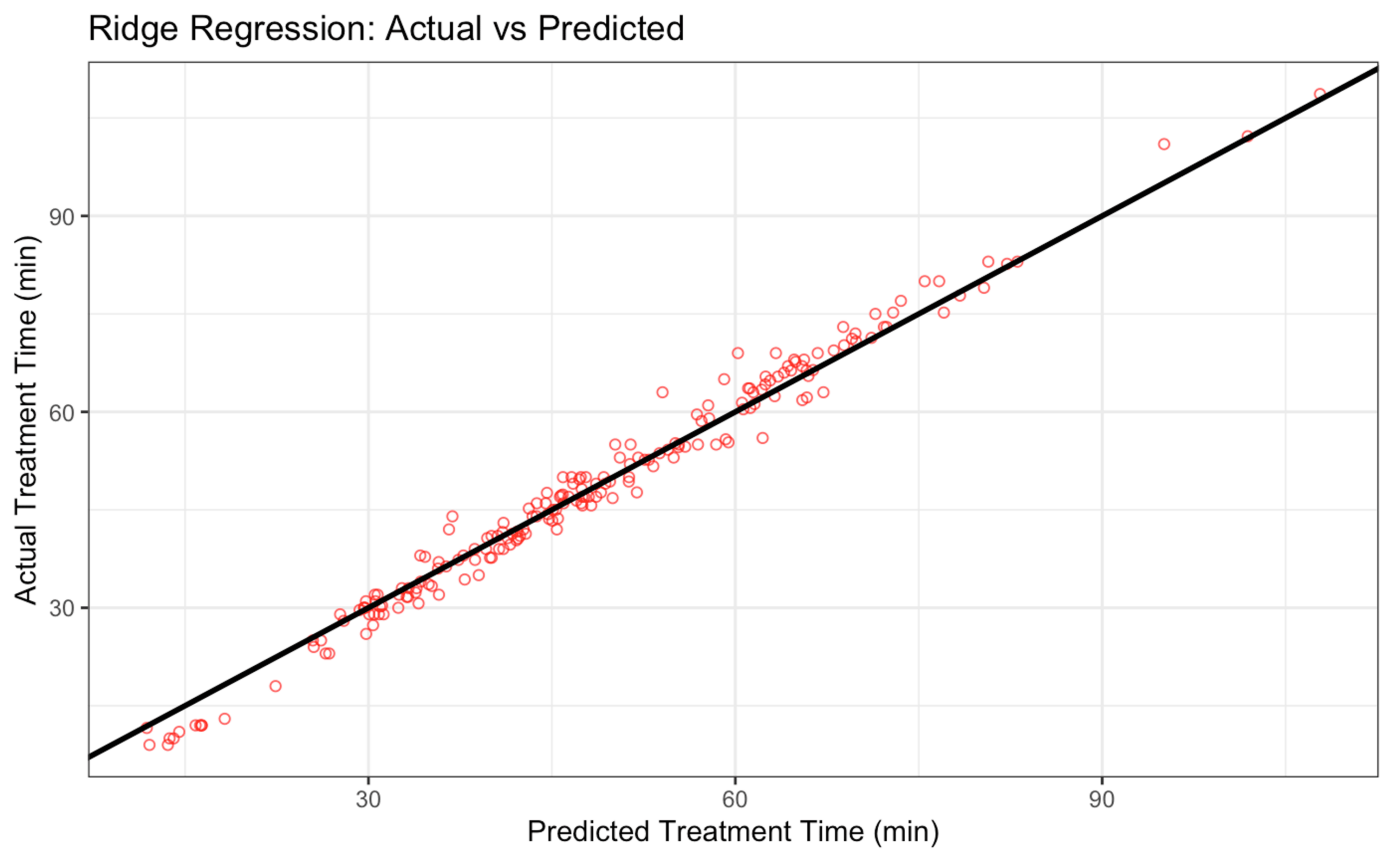
Predicted treatment time versus actual treatment time using the final Ridge Regression model. The close alignment with the identity line (black) indicates high model accuracy, with predictions typically within two minutes of the true duration. Image Credit: Akil Anthony

To build a usable predictive equation, we applied Ridge Regression (Figure 3). Ridge Regression is a modified form of linear regression that adds a penalty to large coefficients, which helps stabilize the model when predictors are correlated [14]. In our dataset, many variables (like setup time, gantry time, table time, kV imaging and processing time, and Linac time) were moderately correlated, so using ordinary linear regression would risk overfitting or unstable estimates. Ridge Regression is especially well-suited for (i) Multicollinearity: It avoids issues when predictors convey overlapping information and (ii) Interpretability: Unlike black-box models, Ridge Regression still outputs a linear equation that clinicians can understand and apply. The following predictors were used: (i) Target number, (ii) Setup time, (iii) Number of isocenters, (iv) Gantry time, (v) Pathology, (vi) Dose, (vii) Number of beams, (viii) Fractions, and (ix) Prescription isodose line.

**Figure 4:**
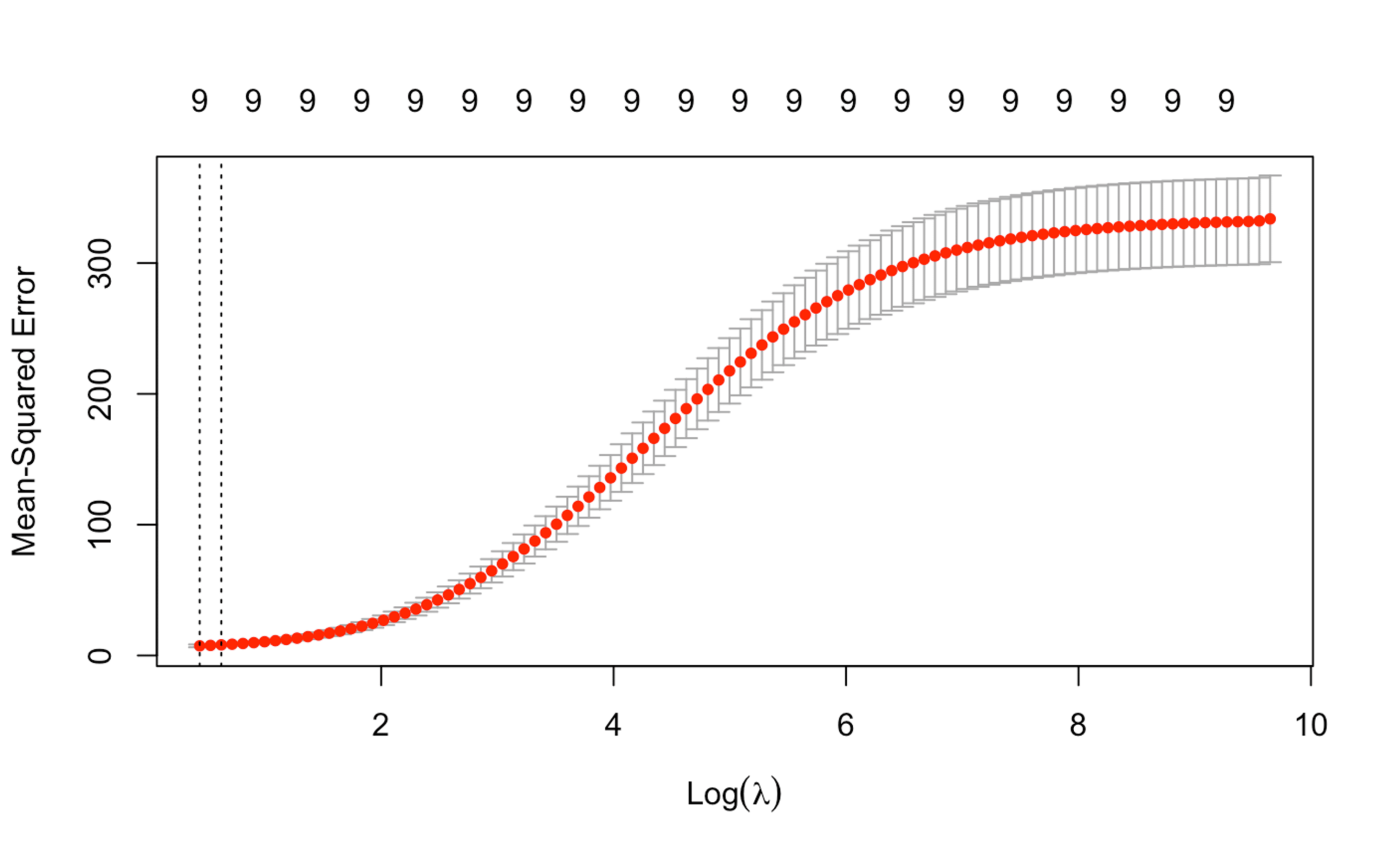
MSE plotted against log(λ) during 10-fold cross-validation for Ridge Regression. The minimum MSE corresponds to the optimal penalty value (λ), which balances model complexity and generalizability. Image Credit: Akil Anthony

We used 10-fold cross-validation to tune the regularization parameter (λ) (Figure 4). Cross-validation is a statistical method used to evaluate how well a predictive model generalizes to an independent dataset. In other words, it helps us estimate how accurately a model will perform on unseen data, which is especially important in clinical settings where overfitting can lead to inaccurate predictions for new patients. In our study, we used 10-fold cross-validation: (i) The dataset is randomly split into 10 equal parts, or "folds", (ii) The model is trained on 9 of those folds and tested on the remaining one, (iii) This process repeats 10 times, each time using a different fold as the test set, and (iv) The average performance metrics (like R², RMSE, and MAE) across all 10 runs are calculated to give a more robust estimate of model accuracy.

Model performance metrics

R² = 0.984: This means that 98.4% of the variation in actual treatment time is explained by the model. In clinical terms, the model is extremely effective at estimating how long a patient's treatment will take.

RMSE = 2.49 minutes: This measures how far off the model is from the actual value, giving extra weight to larger errors. A lower RMSE means more consistent predictions with fewer large misses.

MAE = 1.94 minutes: The model is, on average, within 1.94 minutes of the actual treatment time - far more precise than ZAP-X’s built-in estimate.

Final Ridge Regression equation

Predicted treatment time (minutes) = 9.88790526 + 1.31426515⋅target number + 0.91186794⋅setup time + 0.80920240⋅number of isocenters + 0.75972393⋅gantry time + 0.19585445⋅pathology + 0.07933682⋅dose + 0.04807348⋅number of beams − 1.94623751⋅fractions − 0.05602352⋅prescription isodose line

To employ this equation, the average setup time and gantry time were used. The average setup time and gantry time for 200 patients treated at JSUMC were 11 ± 12 minutes and 30 ± 10 minutes, respectively.

Residual analysis

**Figure 5:**
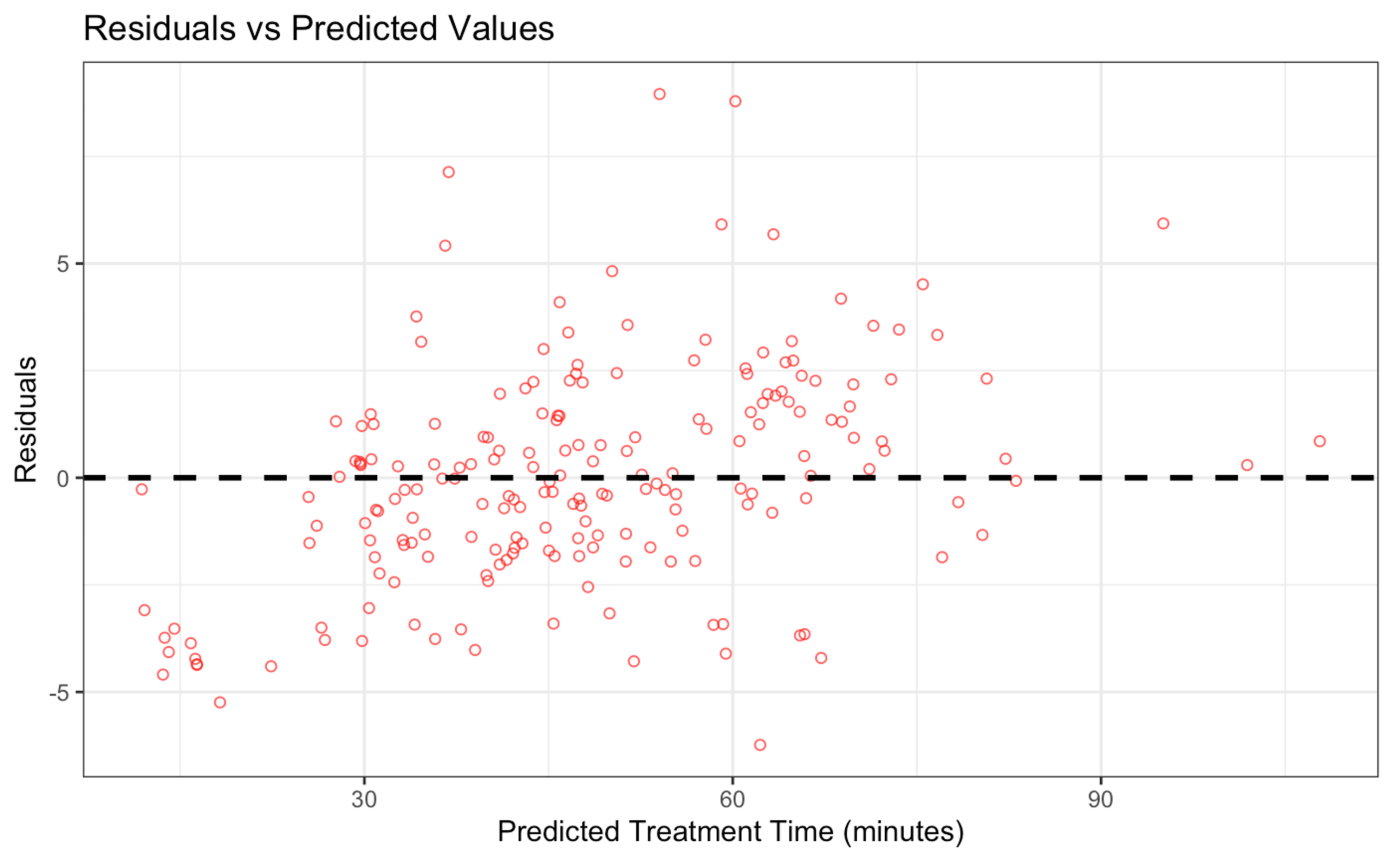
Plot of residuals (actual – predicted) against predicted treatment time for the Ridge model. The residuals are randomly scattered around zero with no clear pattern, indicating a good fit with no major bias across prediction ranges. Image Credit: Akil Anthony

Residuals, the difference between predicted and actual treatment time, were evenly distributed around zero and showed no trends (Figure 5). This indicates that the model is not systematically over- or under-predicting for specific patient types and confirms that key sources of variability were captured by the model.

## Discussion

This study demonstrates that the ZAP-X system’s internally calculated treatment time consistently underestimates the true time patients spend undergoing SRS. Our analysis reveals that this underestimation is largely due to the system’s assumption of optimal workflow conditions, especially regarding setup time, which in practice varies significantly from patient to patient.

Through Random Forest variable importance analysis, we found setup time to be the most predictive feature for actual treatment duration, more influential than treatment planning variables like dose or number of beams. This indicates that non-dosimetric, procedural components of care play a disproportionately large role in determining how long treatment actually takes. Clinically, this makes intuitive sense: patients with different body sizes, mobility levels, or levels of cooperation can require vastly different amounts of time to position, immobilize, and image before treatment begins.

Despite this, the ZAP-X system currently assumes a constant 10-minute setup time for each patient according to the vendor. Our results show that this is not a trivial oversight - ignoring setup variability introduces significant prediction error. Our Ridge Regression model directly addresses this gap. By using readily available treatment planning variables, including setup time, it predicts treatment time with 98.4% accuracy and an average error under two minutes. Unlike Random Forests or other black-box algorithms, Ridge Regression produces an interpretable linear equation that clinicians can apply manually or embed into existing scheduling tools.

While this patient-level model provides a strong baseline, several extensions can make the prediction process even more robust and adaptive: (i) Develop a session-level model: Since some patients undergo multiple treatment sessions (fractions), modeling individual sessions could capture subtle variations across treatment days, such as changes in positioning efficiency, patient condition, or imaging alignment issues. This would allow clinics to adjust daily workflow based on fraction-specific expectations rather than a static per-patient average; (ii) Validate across institutions: While our dataset comes from the most experienced ZAP-X site in the United States, different institutions may have unique workflows, staff expertise levels, or patient populations. Testing the model’s performance in other centers would confirm its generalizability and allow for site-specific calibration if needed; (iii) Integrate into ZAP-X’s planning software: Embedding the model directly into ZAP-X or its companion software would enable real-time predictions during treatment planning. This would automate time estimation and reduce manual entry, creating a seamless clinical tool for physicians, dosimetrists, and radiation therapists; (iv) Update modeling: The ZAP-X is a constantly evolving system and the regression model presented in this study captures up to the latest system version (DP-1010). Future updates are planned for the system which may significantly affect the treatment time which will require updating the model. According to the vendor, a gyroscopic correction update for the ZAP-X system will be implemented which may help reduce setup time. The gyroscopic correction will use the gantry to make automatic corrections for rotational offsets (pitch, roll, and yaw) to enhance precision upon patient misalignment [15]. The second greatest factor contributing to the treatment time was the gantry time. Currently, the ZAP-X utilizes static beams. According to the vendor, an arc treatment update is planned which will reduce the gantry time significantly [16].

This project not only quantifies why ZAP-X’s treatment time estimates are inaccurate but also offers a concrete solution that is ready for real-world use. By identifying and modeling the overlooked procedural components of SRS, we pave the way for smarter, more responsive treatment systems that align with the real-time demands of patient care. 

Several limitations should be considered in this study on analyzing ZAP-X treatment time. The study was conducted at a single institution, limiting the generalizability of findings. Other institutions with different patient populations, workflow efficiency, staff expertise, operational setups, and equipment may encounter varied results. The Ridge Regression model was trained and tested retrospectively with internal cross-validation. There is no external or prospective validation on new patient cohorts to confirm real-world performance. 

## Conclusions

In this study, we developed and validated a predictive model for ZAP-X treatment time using regression techniques, demonstrating significant improvements over the system's built-in time estimates. Our analysis revealed that the ZAP-X system consistently underestimates actual treatment time, primarily due to its static assumptions about setup time. Through Random Forest analysis, we identified setup time and gantry time as the most critical factors influencing treatment duration, followed by number of beams and number of isocenters. By employing Ridge Regression, we constructed a robust predictive model that explained 98.4% of the variance in treatment time, achieving an average prediction error of less than two minutes. This model not only outperformed the ZAP-X’s internal estimates but also maintained interpretability, making it practical for clinical use. Our findings underscore the importance of accounting for procedural factors, particularly patient setup time, in accurately predicting ZAP-X SRS treatment durations.
